# Fast and Forceful: Modulation of Response Activation Induced by Shifts of Perceived Depth in Virtual 3D Space

**DOI:** 10.3389/fpsyg.2016.01939

**Published:** 2016-12-15

**Authors:** Thorsten Plewan, Gerhard Rinkenauer

**Affiliations:** Department of Ergonomics, Leibniz Research Centre for Working Environment and Human Factors (IfADo)Dortmund, Germany

**Keywords:** reaction time, response force, depth perception, 3D, urgency, size constancy

## Abstract

Reaction time (RT) can strongly be influenced by a number of stimulus properties. For instance, there was converging evidence that perceived size rather than physical (i.e., retinal) size constitutes a major determinant of RT. However, this view has recently been challenged since within a virtual three-dimensional (3D) environment retinal size modulation failed to influence RT. In order to further investigate this issue in the present experiments response force (RF) was recorded as a supplemental measure of response activation in simple reaction tasks. In two separate experiments participants’ task was to react as fast as possible to the occurrence of a target located close to the observer or farther away while the offset between target locations was increased from Experiment 1 to Experiment 2. At the same time perceived target size (by varying the retinal size across depth planes) and target type (sphere vs. soccer ball) were modulated. Both experiments revealed faster and more forceful reactions when targets were presented closer to the observers. Perceived size and target type barely affected RT and RF in Experiment 1 but differentially affected both variables in Experiment 2. Thus, the present findings emphasize the usefulness of RF as a supplement to conventional RT measurement. On a behavioral level the results confirm that (at least) within virtual 3D space perceived object size neither strongly influences RT nor RF. Rather the relative position within egocentric (body-centered) space presumably indicates an object’s behavioral relevance and consequently constitutes an important modulator of visual processing.

## Introduction

In everyday life the visual system is confronted with a vast majority of ambiguous information. Interacting with a three-dimensional (3D) environment for instance requires a precise identification of locations in depth associated with different objects. However, in the past the mechanisms underlying visual perception and attention have often been investigated using two-dimensional (2D) stimulus material, while only a minority of studies was concerned with the impact of 3D stimulus material (e.g., Downing and Pinker, 1985; Andersen, 1990; Andersen and Kramer, 1993; Theeuwes et al., 1998; Theeuwes and Pratt, 2003). The latter issue recently received more attention and was addressed by an increasing number of investigations (e.g., Bauer et al., 2012; Chen et al., 2012; Finlayson et al., 2013; Weidner et al., 2014; Finlayson and Grove, 2015; Wang et al., 2015, 2016; Jiang et al., 2016; Papenmeier and Schwan, 2016; Plewan and Rinkenauer, 2016). Some of these studies imply that perceptual mechanisms do not always correspond between 2D and 3D settings. For instance, it has been known for a long time that there is an inverse relationship between stimulus intensity or size and simple reaction time (RT; Teichner and Krebs, 1972; Cattell, 1886). Using 2D stimuli there is growing evidence that faster RT is related to perceived rather than physical stimulus properties (Sperandio et al., 2010; Plewan et al., 2012; Savazzi et al., 2012). In a related study, Sperandio et al. (2009) presented stimuli on a computer screen while altering the viewing distance between screen and observer. According to the principles of size constancy (e.g., Epstein et al., 1961; Sperandio and Chouinard, 2015) perceived size remains constant while at the same time distance to an object increases and physical (i.e., retinal) size decreases. In contrast, keeping retinal size constant across depth planes leads to an increase in perceived size in more distant locations. In line with these considerations Sperandio and colleagues reported constant RT as long as perceived stimulus size was kept constant across depth planes, while participants indeed reacted faster in response to target stimuli that were perceived as larger in the most distant depth location. However, the observed effects were limited to renderings of familiar object stimuli (i.e., a tennis ball compared to plain disks) and in an additional experimental condition without depth and distance information participants’ response behavior was solely affected by retinal object size.

This relationship between RT and perceived object size has lately been challenged in a series of experiments in which stimuli were presented in virtual 3D space (Plewan and Rinkenauer, 2016). Spherical objects were displayed stereoscopically via head-mounted displays (HMD) such that the target could be displayed in three different depth planes (near, midway, far). At the same time retinal target size was modulated similar to the procedure outlined by Sperandio et al. (2009). In contrast to these previous results no faster RT was observed when retinal size was kept constant across depth planes (i.e., increasing perceived stimulus size). Quite the contrary, RT was faster for targets presented closer to the observers, irrespective of retinal size modulation.

Regarding their behavioral implications the latter findings appear plausible as objects in closer proximity to an observer often require immediate action or even constitute a serious threat (Graziano and Cooke, 2006). There are a number of empirical findings that strengthen this notion. For instance, it has been shown that a capture of (visual) attention does not occur for all dynamic stimuli: In order to simulate object motion size was decreased (receding condition) or increased (looming condition). Only in the latter condition a capture of attention was observed while receding stimuli failed to do so (Franconeri and Simons, 2003). However, there are also contrasting results showing attentional capture by receding stimuli (Abrams and Christ, 2005). Abrams and Christ presented their stimuli material stereoscopically and found that receding stimuli indeed were able to capture attention. According to the authors it is the onset of motion rather than its direction that conveys this effect. Subsequently, there was agreement that the onset of motion in particular might not be necessary but yet seems to be beneficial to capture attention (Franconeri and Simons, 2005; Abrams and Christ, 2006). Based on this line of research Franconeri and Simons (2003, 2005) formulated the behavioral urgency hypothesis stating that approaching stimuli receive processing priority over other stimuli. In line with that it has recently been reported that in visual search tasks there seems to be a kind of egocentric search gradient through space (Finlayson and Grove, 2015). The authors asked participants to search for targets across different depth planes and found that this search is completed faster if a target is located closer to the participants. Attentional reorientation in virtual 3D space has also been shown to be differentially modulated in near and far space, respectively. For instance, using an endogenous attentional cuing task it was revealed that reorienting to targets unexpectedly appearing closer to an observer are faster than to targets which are located farther away (Chen et al., 2012). In a similar study, Wang et al. (2016) employed an exogenous attentional cuing task and found common inhibition of return effects only for targets presented in near space.

The findings outlined above are derived from the measurement of response times and error rates. However, recording response force (RF) has been established as an additional parameter to investigate sensorimotor processes (Giray and Ulrich, 1993; Ulrich et al., 1998; Miller et al., 1999; Jaśkowski et al., 2000; Stahl and Rammsayer, 2005). For instance, Ulrich and colleagues disentangled the impact of stimulus duration and intensity on simple RT. The authors asked their participants to respond as fast as possible to auditory stimuli. As expected participants responded faster (decreased RT) and more forceful (increased RF) when stimulus duration or intensity was increased. While RT did not further decrease with stimulus durations lasting longer than 80 ms, RF was still susceptible to stimulus durations over 300 ms (Ulrich et al., 1998). Likewise, Stahl and Rammsayer (2005) revealed that the time windows in which RT and RF can be modulated by accessory stimulation substantially differ with that one associated to RF being much longer. Even though RT and RF effects often point into the same direction both measures are not redundant and usually uncorrelated across trials (Giray and Ulrich, 1993; Ulrich and Mattes, 1996). This denotes the effect that fast responses do not necessarily coincide with forceful responses, and vice versa. Besides RF has also been shown to be not only sensitive to differences of stimulus properties but also can be affected by task demands. For instance, RF is susceptible to experimental variations of time pressure (Rinkenauer et al., 2004). Rinkenauer et al. (2004) varied the target response interval and found that shorter intervals were associated with more forceful responses. In particular, the study revealed that RF and the duration of late motor processes – indicated by response logged lateralized readiness potential – were affected differently by time pressure manipulation. Furthermore, there is evidence from a study conducted in a driving context that participants can successfully employ brake force as sensitive measure of perceived urgency (Lewis et al., 2013). As outlined above closer objects might be considered as behaviorally urgent. Thus, it is likely to assume that RF constitutes a suitable aid to further investigate the mechanisms of perceived size and urgency of objects along different depth planes.

Accordingly, in the present study measures were recorded via a force-sensitive key in order to determine RF and RT simultaneously. Otherwise the experimental design was adopted from a similar study reported by Plewan and Rinkenauer (2016): Participants had to perform a simple reaction task, i.e., they had to react as fast as possible in response to spherical objects which were presented in virtual 3D space while retinal size was either constant or altered across depth planes. In addition, the offset (small vs. large) between both target depth planes was also varied in two separate experiments. Based on previous results it was hypothesized that RT will be shorter for targets in closer proximity to the observers, irrespective of retinal size modulation. This would be in agreement with the assumption of a body-centered (attentional) search gradient through space. At the same time RT advantage for closer objects is expected to be even higher if the offset between target locations becomes larger. Assuming that the behavioral urgency triggered by a target is a function of target position one can expect a more pronounced RT difference if the offset between targets is increased. Thus, with respect to Experiment 1 the offset between target depth planes was further increased in Experiment 2 while keeping all other experimental parameters constant. This way Experiment 2 was intended to test whether increased distance between observer and target indeed leads to enlarged RT effects and moreover serve to validate the data pattern of Experiment 1. Similar predictions can be made for RF. In relation to the behavioral urgency hypothesis RF can be taken as an indicator for response activation. Thus, one may expect a more intensive response activation for near than for far objects. Again, this effect should be even more pronounced if the offset between target depth planes is increased. As a further experimental variable a familiar object (i.e., a soccer ball) was introduced as target in addition to a plain white sphere. In their study Sperandio et al. (2009) emphasized that RT modulations to differences in perceived size might be limited to familiar objects. The absence of any RT effects related to perceived size modulation in previous experiments maybe was related to this circumstance. Hence, it was further hypothesized that the type of target (i.e., sphere vs. ball) will have an impact on RT.

## Materials and Methods

Two experiments were conducted based on the experimental design recently introduced by Plewan and Rinkenauer (2016). Both experiments were identical with only the offset between target depth positions (see below) altered between experiments. A new and independent sample of participants was tested for each experiment.

### Participants

In total 41 participants (31 female, 19–37 year) were recruited. According to the Edinburgh handedness inventory (Oldfield, 1971) four of them were left-handed. All participants had normal or corrected-to-normal vision and reported no history of psychiatric or neurological disorders. Participants received either a compensation (10 €/h) or course credit. The experiment was conducted in accordance with the declaration of World Medical Association (2013) and all participants gave written informed consent. The experimental framework was approved by the Ethics Committee of the Leibniz Research Centre for Working Environments and Human Factors. Prior to the actual experiment stereo vision capability was verified using TNO (Netherlands Organization for Applied Scientific Research) test for stereoscopic vision (all participants revealed stereo-thresholds of < = 120 arc sec). There were technical problems that corrupted the data from two participants and another participant chose not to finish the experiment. Five additional participants were excluded from further analyses due to low task performance (i.e., >30% false alarms). Thus, the sample for Experiment 1 consisted of 14 participants (11 female, 20–27 years, one left-handed), while data from 19 participants was included in the analysis of Experiment 2 (14 female, 19–37 years, two left-handed). Participants responded with their preferred hand.

### General Procedure and Experimental Design

The experimental setup was generated using the virtual reality software Vizard 4 (©WorldViz, LLC). Stimulus material was presented via professional stereo head-mounted displays (HMD, nVisor ST50), with a resolution of 1280 × 1024, a refresh rate of 60 Hz (single frame rate 16 ms) and a 50° diagonal field-of-view. The visual focus of the HMD was set to 10 m. Both screen displays are arranged in a way such that they are placed closely in front of the participants’ eyes. Therefore, a vivid depth impression can be evoked via stereoscopic presentation. Responses were recorded using a custom made force-sensitive key device. The key device basically consists of a small rectangular metal bar representing the response button (Giray and Ulrich, 1993). Any force applied to this bar results in a measurable electrical signal which was digitized with 1000 Hz. A force of 100 centinewton (cN) corresponded to a voltage deflection of about 0.250 mV. Forces exceeding a threshold of 400 cN were registered as responses.

**FIGURE 1:**
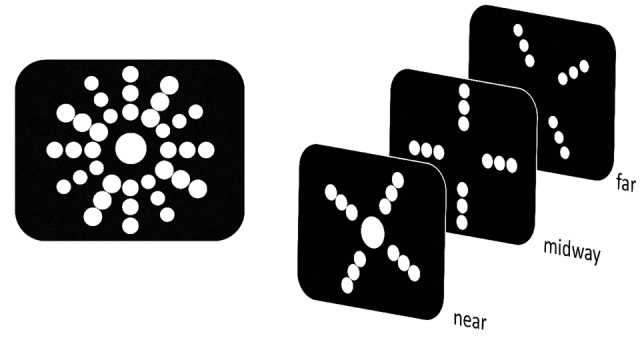
**Schematic illustration of the stimulus material used in both experiments.** Left part depicts a front view as seen by the participants via head-mounted displays (HMD). Right part portrays the stimulus distribution across depth planes. The central target either appeared in the near or far depth plane. Surrounding spheres were visible throughout the whole experiment in order to create a reference space for the target. Offset between near and far depth plane was altered between Experiments 1 and 2 (see “Materials and Methods”).

In both experiments either a plain white sphere (henceforth sphere target) or a soccer ball (henceforth ball target) served as target. As soccer is the most popular sport in Germany and omnipresent in television and media it is almost certain that participants had a vivid mental representation of a soccer ball. The target was always displayed in the center of the display and participants were accordingly asked to fixate in the center throughout the experiment. Eye movements were not monitored as dedicated hardware for the employed HMD was unavailable. The actual depth position of the target was determined on a trial-by-trial basis. This way the target was rendered randomly either in near or far position (Experiment 1: 1.14 m vs. 1.42 m; Experiment 2: 1.00 m vs. 1.56 m) with respect to the observer. The target was surrounded by additional 36 spheres presented in empty black space. The additional spheres were arranged in form of three rings (each constituted by 12 spheres) around the central target, with a radius of 18, 27, and 36 cm, respectively (**Figure 1**). In general, relative depth information has been discussed to be an important source for depth perception (Neri et al., 2004; Chopin et al., 2016). Within a virtual 3D setting relative depth information has also been shown to be a prerequisite to detect RT effects (Plewan and Rinkenauer, 2016). Thus, depth location of the surrounding spheres was allocated in a clockwise manner from near to midway to far position (i.e., 1.14, 1.28, 1.42 m [Experiment 1] or 1.00, 1.28, 1.56 m [Experiment 2]) in order to constitute a reference space. This circular reference space was fixed and visible throughout the whole experiment (**Figure 1**). It has been suggested that objects in different regions of 3D space are differentially processed (Previc, 1998). To rule out any potential confounder related to position in 3D space, all stimuli in this study were presented within peripersonal space (i.e., the region immediately surrounding the body; <2m).

An experimental trial was initiated by an auditory cue (presented for 250 ms via build-in headphones of the HMD device). Subsequently, the target followed 500–1000 ms after cue offset and remained on the displays for 100 ms. In addition, catch trials (i.e., trials without target) were included in order to prevent a general response tendency. Participants were instructed to react as fast as possible to target onset while avoiding anticipations or false alarms on catch trials. Erroneous responses on catch trials, as well as anticipatory (too fast, <100 ms) reactions or misses (no/subthreshold reaction within 2000 ms) caused the appearance of the German word “Fehler” (i.e., error) in red letters at the bottom of the display for 500 ms.

Both experiments consisted of a 2 [retinal size (R): constant, variable] × 2 [depth position (D): near, far] × 2 [target type (T): sphere, ball] design. Retinal size was manipulated in two separate blocks (within-subject) while offset between depth positions was modulated as a between subject variable (Experiment 1 vs. Experiment 2). Thus, across all experimental conditions the target’s depth position was (pseudo-) randomly allocated to one of both potential depth positions (near vs. far). In the *retinal size variable* condition the target size was scaled according to size-distance invariance hypothesis: Visual angle decreased proportional with increasing depth. Likewise in the *retinal size constant* condition the physical target size was kept constant across depth planes, in other words, visual angle was not altered between depth positions. According to the manipulation of retinal size the target stimulus subtended 4° (near) and 3.22° (far) visual angle in Experiment 1 or 4.58° (near) and 2.94° (far) in Experiment 2 during *retinal size variable* condition and invariable 3.58° visual angle (Experiment 1 and Experiment 2) across all depth planes in *retinal size constant* condition. Due to stereoscopic presentation the perceived stimulus size was constant across depth planes in *retinal size variable* condition or increasing in *retinal size constant* condition from near to far position. The binocular disparity between the near and far depth planes was about 38.16 arc min in Experiment 1 and 79.07 arc min in Experiment 2. Surrounding spheres subtended about 1.79° visual angle in midway depth plane. Consequently the size in near and far depth plane was 2.01° and 1.61° (Experiment 1) or 2.29° and 1.47° (Experiment 2), respectively.

Both experiments comprised two blocks which in turn contained 600 trials (400 target trials + 200 catch trials). Target location (near, far) was randomly allocated on a trial-by-trial basis. Target Type (sphere, ball) was also individually randomized and experimental blocks were assigned in counterbalanced order (i.e., half of the participants began with *retinal size constant* condition, while the other half conducted *retinal size variable* condition first). Accordingly, each participants completed 1200 trials in a single session, with a self-paced break halfway through each block and between both blocks. Overall, the experiment took about 1 h.

### Analysis

In order to examine simple RT erroneous trials and extreme responses (RT < 100 ms and RT > 1000 ms) were excluded from further analysis. For each valid trial the peak force amplitude (i.e., maximal RF) was determined and single trial RT was defined as the time between target onset and the moment when 25% of the trial’s peak force was exceeded. The corresponding values were used to calculate a mean RT for each experimental condition. This resulted in eight individual parameter per participant (2 retinal size conditions × 2 depth positions × 2 target type) which were subsequently subjected to a repeated measures analysis of variances (ANOVA). Likewise, force measures from all valid trials were employed to determine mean RF for each experimental condition. Again, eight individual mean RF values per participant were computed and these parameters were subjected to a repeated measure ANOVA. Resulting *F*-values, *p*-values, and generalized eta squared (η_G_^2^) are reported (Olejnik and Algina, 2003; Bakeman, 2005). In case multiple comparisons were conducted via *post hoc t*-tests corresponding *t*-values, Cohen’s *d* (Cohen, 1988) and adjusted *p*-values (Benjamini and Yekutieli, 2001) are reported.

## Results

### Experiment 1

Error rates (missing responses, anticipations, and false alarms in catch trials) indicated that participants performed well on the simple reaction task. Approximately 1.45% (range 0–4.33%) of trials across *retinal size variable* condition were erroneous and 1.89% (range 0–4.17%) of trials accounted for errors in *retinal size constant* condition. In addition to the overall low error rates the amount of errors (false alarms and misses) and anticipations was roughly the same. Thus, error rates were not further investigated.

**FIGURE 2:**
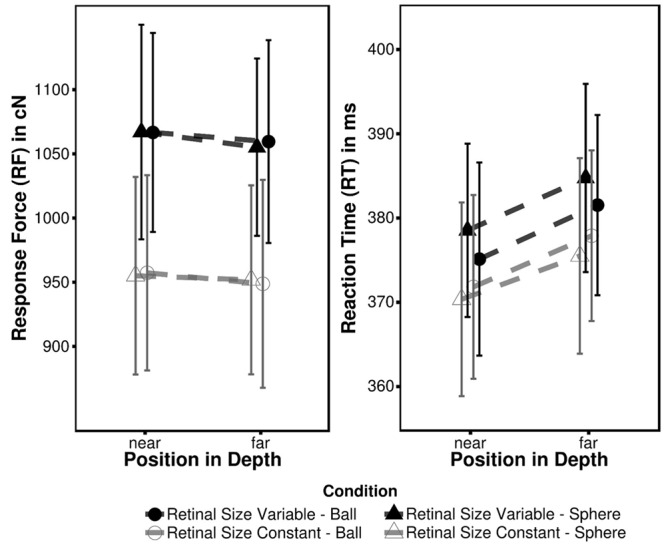
**Mean response force (RF) and reaction time (RT) observed in Experiment 1.** Error bars represent within-subject 95% confidence intervals (Moray, 2008).

Mean RF and RT for each condition are summarized in **Table 1**; **Figure 2**. The 2 × 2 × 2 ANOVA performed on force data revealed a significant main effect of Depth [*F*_D_(1,13) = 4.93, *p* = 0.045, η_G_^2^ = 0.00012] indicating more forceful responses associated with near (1011.66 cN; pooled across retinal size and target type condition) as opposed to far targets (1003.97 cN). The remaining main effects (retinal size condition and target type) as well as all interactions did not reach significance (all *F* ≤ 2.71; all *p* ≥ 0.124). Even though visual inspection of the data gives the impression that there is an RF effect between retinal size conditions this is rather inconsistent across participants and hence non-significant [*F*_R_(1,13) = 2.71, *p* > 0.123]. Most likely these variations in RF can be attributed to a low level of tactile (and/or acoustical) feedback. In contrast to a conventional keyboard or button the force-sensitive key has no terminal position and hence provides only little feedback on current response state. This instance most likely prevented some participants to exhibit a constant force level across both experimental blocks.

**Table 1:** Mean response force (RF) in centinewton (cN) and reaction time (RT) in ms as observed in Experiment 1.

		Retinal size variable		Retinal size constant	
		RF [cN]	RT [ms]	RF [cN]	RT [ms]
Near	Sphere	1067.16 (382.20)	378.52 (51.76)	955.19 (319.31)	370.34 (36.36)
	Ball	1066.83 (383.70)	375.11 (47.93)	957.45 (325.26)	375.48 (39.38)
Far	Sphere	1055.33 (376.66)	384.73 (50.97)	951.97 (314.85)	371.81 (39.69)
	Ball	1059.67 (381.08)	381.52 (53.24)	948.89 (324.16)	377.88 (36.98)

*Values in brackets denote standard deviations (*SD*).*

An equivalent ANOVA performed on RT data points into a similar direction. There was also a significant main effect of depth [*F*_D_(1,13) = 13.11, *p* < 0.001, η_G_^2^ = 0.0047] with near targets (373.94 ms) eliciting faster RT than far targets (379.90 ms). Besides, no other significant main effects or interactions (all *F* ≤ 3.48; all *p* ≥ 0.085) were observed. In spite of significant main effects of depth in the RT and RF data these effects are uncorrelated (all *r* ≤ 0.41, *t* ≤ 1.58, *p* ≥ 0.140). Consequently both measures are not directly linked to each other, indicating that fast responses not necessarily coincide with high RF and vice versa.

The results are in line with previous findings which indicate that targets in closer proximity to an observer elicit faster responses (Plewan and Rinkenauer, 2016). Moreover, a similar effect was associated with RF, as closer objects lead to more forceful responses. However, these effects for RT and RF are independent of (retinal) size modulation and type of target. At least for RT opposite effects have previously been demonstrated (Sperandio et al., 2009). In their study Sperandio and colleagues presented targets of identical retinal size which differed in terms of perceived size (small vs. large). The reported data indicate that perceptually larger targets elicited faster RT even when presented in a more distant location. This is at odds not only with the present findings but also with the assumption that closer targets indicate a higher behavioral urgency which in turn triggers faster responses. However, the latter assumption would also imply larger effects on RT (and likewise on RF) if distance between target depth planes is further increased. Therefore, in order to validate the present findings and further investigate the role of target depth a second experiment was performed. Accordingly, Experiment 2 was essentially identical to Experiment 1 with the only difference that the offset between target locations in depth was increased (see “Materials and Methods”).

### Experiment 2

Again, error rates (missed responses, anticipations, and false alarms in catch trials) indicated high task performance. Approximately 1.77% (range 0–7.83%) of trials across *retinal size variable* condition were erroneous and 1.69 % (range 0–4.17 %) of trials accounted for errors in *retinal size constant* condition. As a result of these low numbers errors rates were not further investigated.

**FIGURE 3:**
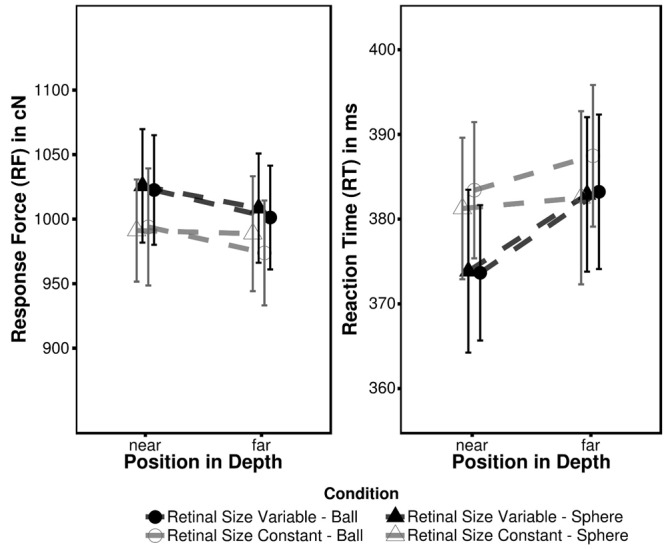
**Mean RF and RT observed in Experiment 2.** Error bars represent within-subject 95% confidence intervals (Moray, 2008).

The mean RF and mean RT for each condition are summarized in **Table 2**; **Figure 3**. The 2 × 2 × 2 ANOVA performed on RF data revealed main effects of Depth [*F*_D_(1,18) = 20.43, *p* < 0.001, η_G_^2^ = 0.00044] and Target Type [*F*_T_(1,18) = 4.84, *p* = 0.041, η_G_^2^ = 0.00006] indicating more forceful responses to closer targets and less intense responses to ball targets as compared to sphere targets (near: sphere = 1008.48 cN; far: 993.20 cN). The main effect of retinal size was not significant [*F*_R_(1,18) = 0.57, *p* = 0.461] as was neither of the interactions (all *F* ≤ 1.56; all *p* ≥ 0.227). As outlined above RF differences between retinal size conditions most likely can be attributed to a shift of individual force level across blocks.

**Table 2:** Mean RF in cN and RT in ms as observed in Experiment 2.

		Retinal size variable		Retinal size constant	
		RF [cN]	RT [ms]	RF [cN]	RT [ms]
Near	Sphere	1025.91 (388.26)	373.87 (63.46)	991.28 (363.76)	381.28 (57.03)
	Ball	1022.70 (392.84)	373.68 (59.86)	994.05 (369.04)	383.43 (55.85)
Far	Sphere	1008.65 (382.83)	382.94 (66.16)	988.85 (365.26)	382.54 (57.70)
	Ball	1001.38 (374.60)	383.25 (64.03)	973.90 (361.76)	387.50 (58.88)

*Values in brackets denote *SD*.*

An equivalent ANOVA on RT also revealed a significant effect of depth [*F*_D_(1,18) = 16.68, *p* < 0.001, η_G_^2^ = 0.0026] and an additional Depth × Retinal Size interaction [*F*_D × R_(1,18) = 5.15, *p* = 0.0360, η_G_^2^ = 0.0008]. Again the former effect denotes faster RT for closer targets (near: 378.07; far: 384.10 ms), while the interaction in particular indicates faster RT in the *retinal size variable* condition, when the target (irrespective of type) is presented in near position (**Table 2**). To further disentangle this effect four (2 retinal size × 2 target type conditions) independent paired two sample *t*-tests were conducted between near and far target position. In the *retinal size variable* condition the effects indeed were more pronounced as both target types led to faster responses in near position [sphere: *t*(18) = -2.79, *p* = 0.050, *d* = 0.64; ball: *t*(18) = -4.84, *p* = 0.001, *d* = 1.11], while this was not the case in *retinal size constant* condition [sphere: *t*(18) = -0.46, *p* = 1.00, *d* = 0.10; ball: *t*(18) = -2.03, *p* = 0.160, *d* = 0.46]. The main effect of Target Type only approached significance [*F*_T_(1,18) = 4.14, *p* = 0.057, η_G_^2^ = 0.00024] whereas the effect of Retinal Size was not significant [*F*_R_(1,18) = 0.51, *p* = 0.484] as well as the remaining interactions (all *F* ≤ 2.33; all *p* ≥ 0.144). As recognized in Experiment 1, although RT and RF were similarly affected by depth modulation the corresponding values were uncorrelated (all *r* ≤ 0.42, *t* ≤ 1.91, *p* ≥ 0.074).

The findings are in line with those observed in Experiment 1. Increasing the offset between near and far target position led to a more pronounced RT advantage for closer targets as well as to stronger RF. However, additional ANAOVAs on both dependent variables incorporating target offset as between-subject factor failed to strengthen this observation statistically. In fact there were no main effects or interactions associated with target offset (all *F* ≤ 2.47; all *p* ≥ 0.126). Nonetheless, in contrast to Experiment 1 RF and RT were also differentially modulated by the experimental variations. For instance, using different kinds of targets (sphere vs. ball) only had an effect on RF, while retinal size modulation resulted in a significant interaction of RT.

## General Discussion

The present study was designed to extend the knowledge about the perception of perceived object size and depth in virtual 3D space. Therefore, RF was recorded to assess response activation in addition to RT which has been employed in previous research (Plewan and Rinkenauer, 2016). In two experiments simple RT was measured via a force-sensitive key device while participants had to respond to target objects which were presented in different depth locations within a virtual 3D space. As expected RT was susceptible to changes in targets’ depth positions and the force exerted by the participants was also strongly modulated by target depth. Even though both dependent variables (RT and RF) reveal effects that point into the same direction they were uncorrelated and thus constitute two independent measurements (Giray and Ulrich, 1993; Stahl and Rammsayer, 2005).

There are several reports that emphasize the use of RF in order to investigate visual processing (Giray and Ulrich, 1993; Ulrich and Mattes, 1996; Ulrich et al., 1998; Miller et al., 1999; Jaśkowski et al., 2000; Rinkenauer et al., 2004; Stahl and Rammsayer, 2005). The present experiments underline the notion that RF constitutes a reasonable supplement to RT. Changes in perceived target distance yielded the most pronounced effects on both measures (i.e., closer targets causing faster RT and stronger RF). However, particularly Experiment 2 revealed that RT and RF can also be differentially affected. For instance, with respect to the modulation of Target Type RF was apparently a more sensitive tool to detect differences between ball and sphere targets (see below). In contrast, only analysis of RT data revealed an interaction between retinal size (constant vs. variable) and depth, indicating faster RT in response to closer targets only in the *retinal size variable* condition. Furthermore, there seems to be no correlational relationship between RT and RF although perceived distance clearly has an influence on both variables. This observation is a common finding in studies recording RF and has led to the interpretation that RF and RT represent different aspects of visual processing (Giray and Ulrich, 1993; Stahl and Rammsayer, 2005). Traditionally, in chronometry research RT is regarded to express the final state of information processing (Donders, 1969; Sternberg, 1969; Luce, 1986). Experimental modulations of RF in contrast have been discussed to indicate different levels of bodily arousal or activation (Giray and Ulrich, 1993; Jaśkowski et al., 1995). Accordingly, stronger responses can only be observed if a particular stimulus is able to enhance (motoric) activation. Furthermore, RF might also be related to (motivational) evaluation processes (Puca et al., 2006). In their study Puca et al. (2006), for instance, investigated withdrawing and approaching movements and found that RF was related to participants’ motivational setting.

The present findings support the notion that both measures represent different aspects of visual processing as distinct effects were associated to them. Even though in the past RF has been found to be susceptible to several experimental variations like time pressure (Jaśkowski et al., 2000; Rinkenauer et al., 2004) or stimulus intensity (Ulrich et al., 1998; Miller et al., 1999) to our knowledge this study constitutes the first attempt to reveal a relationship between RF and depth perception. The use of a force-sensitive device in this context was intended to provide deeper insight into the processes underlying the responses behavior. However, the contribution of RF was limited in this regard as the differences between RF and RT overall were relatively small. Most observed effects point into the same direction and RT effects were more pronounced across both experiments. This observation might be related to the employed experimental paradigm. Performing a simple reaction task requires only little cognitive assessment of the stimuli. Therefore, it is conceivable that effects of RF will be more pronounced in paradigms that involve categorical judgements like go-nogo or alternative-forced choice tasks.

Measuring RF was also associated with much more variability when compared to RT. Therefore, the observed effect sizes are relatively small as much of the variance accounts for this individual differences of RF between experimental blocks. This circumstance most likely is related to the uncommon key device: Using a conventional keyboard there is a terminal position of the key and pressing the key is usually accompanied by a specific sound. By this means one can infer the amount of isotonic force as well as the current response level. The force-sensitive key used in the present experiments in contrast requires the application of isometric force and thus provides no feedback to the participants, which might hinder some of them to keep a constant response level across both experimental blocks. It could be helpful for further investigations to familiarize participants with the key device and determine the maximal force for each participant individually. This way relative measures of RF could be compared and the impact of large (inter-)individual differences would be attenuated.

### Perceived Depth

As observed in previous research the target’s perceived depth location strongly influenced RT. Even though there are some results that do not confirm this observation (e.g., Theeuwes et al., 1998; Dent et al., 2012; Finlayson et al., 2012) there is a growing amount of reports revealing that targets presented closer to an observer result in faster RT (e.g., Shulman et al., 1979; Downing and Pinker, 1985; Gawryszewski et al., 1987; Chen et al., 2012; Finlayson and Grove, 2015; Plewan and Rinkenauer, 2016; Wang et al., 2016). However, comparisons of different studies on 3D perception are particularly difficult as those studies employ distinct techniques to induce depth impression. Furthermore, it plays an important role whether stimuli are manipulated in virtual or real 3D space. Disparity, for instance, is the only strong depth cue when stimuli are presented via HMD. In contrast, if stimuli are displayed on a screen (e.g., together with shutter glasses) or even integrated in real 3D settings several (monocular) depth cues like relative size, accommodation, or motion parallax may be available. Those depth cues are inherent to the displayed stimuli and cannot experimentally manipulated. Therefore, even subtle differences between 3D settings may alter (3D) perception substantially. Irrespective of this, the present finding indicate that closer targets not only elicit more forceful responses but also lead to faster RT. This supports the view that closer objects may receive processing priority. In spite of a larger variability across experimental blocks associated to RF both measures (RT and RF) indicate a consistent difference between targets in near and far location within participants. Numerically the modulation of RF and RT even seemed to be stronger pronounced in Experiment 2 which comprised a larger offset (i.e., increased perceived distance) between near and far target location. However, the increase of target offset was not related to any statistical effects (albeit target offset was only included as a between subject factor in the present experiments). It would be an interesting question for future research whether an impact of target offset within participants can be established.

In a recent study investigating visual search across different depth planes Finlayson and Grove (2015) reported an advantage for targets in near space. When targets and distractors were distributed across depth planes response times decreased when the target was presented in a closer depth plane. The authors suggested that these findings indicate the existence of an egocentric visuospatial (search) gradient spreading from near to far space (Finlayson and Grove, 2015). Although in the present experiments a simple reaction rather than a visual search task was employed, the findings can also be interpreted in favor of a body-centered spatial gradient. If there is a tendency to increase search speed or efficiency in closer proximity this should also hold true for a more fundamental task as employed in the current study. The existence of such a visuospatial gradient in turn would be in agreement with the behavioral urgency hypothesis. According to this theory objects closer to (or approaching) an observer are assumed to elicit faster responses as these stimuli demand instantaneous processing priority (Franconeri and Simons, 2003). It was even possible to identify neural populations in animals and humans that specifically respond to looming objects (Graziano and Cooke, 2006; Preuss et al., 2006; De Vries and Clandinin, 2012). From a behavioral point of view it is plausible that closer objects are associated with a higher urgency and consequently elicit more forceful responses which are quickly executed.

An elaborated model of 3D space (Previc, 1998) makes distinctions between the perceptual mechanisms in peripersonal space (within 2 m from the body) and extrapersonal space (more distant and peripheral parts of the visual field). All stimuli in the present experiments were presented within peripersonal space. Thus, the present results are limited in this regard and it would be interesting to see whether the response pattern would be similar when targets are presented in different regions of 3D space. However, visual stimuli can only be properly fused in a relatively small spatial area (Panum, 1858; Palmer, 1961). Therefore, the experimental framework as employed in the present study might not be suitable to test manipulations of larger shifts in depth. Also there is evidence for selective neural population responding to crossed and uncrossed disparity information (i.e., objects presented in front or behind the horopter; Poggio et al., 1988). Participants were not restricted to a particular depth plane in the present study. Therefore, the current finding are limited in this respect, too. Yet, there are findings suggesting that crossed and uncrossed stimulus properties do not necessarily lead to differential visual processing (Jaschinski and Schroth, 2008). Furthermore, the current results are very similar to a related study in which participants fixated the midway depth plane (Plewan and Rinkenauer, 2016). Still it seems worth to further investigate the role of crossed and uncrossed disparities in (virtual) 3D environments.

### Retinal Size Modulation

Recently, several publications reported that simple manual RT can be modulated by changes in target size and that these effects are strongly related to changes in perceived rather than retinal (physical) size. For instance, this effect has been investigated using visual illusions as stimulus material. In this case targets have identical physical size properties (i.e., *retinal size constant*) but can substantially differ in terms of perceived size. Accordingly those targets that were perceived as larger usually elicited faster RT (Sperandio et al., 2010; Plewan et al., 2012; Savazzi et al., 2012). Likewise it was shown, that RT decreases if retinal object size is kept constant across different depth planes (i.e., the object becomes perceptually larger; Sperandio et al., 2009). Yet, a depth modulation solely based on changes in disparity failed to replicate this interrelation between retinal size modulation and RT (Plewan and Rinkenauer, 2016). The same is true for the present results. Neither experiment revealed significant main effects in RT or RF which were related to variations in retinal stimulus size across depth planes. However, an interaction between retinal size condition and depth was observed in Experiment 2. Increasing the offset between near and far target (Experiment 2 vs. Experiment 1) location apparently had stronger impact in the *retinal size variable* condition which in turn led to faster RT for near targets only in this condition. Again, this finding is in line with the behavioral urgency hypothesis (see above; Franconeri and Simons, 2003) since the farther located target becomes less behavioral relevant. The *retinal size constant* condition in contrast denotes a paradoxical viewing situation: Under natural viewing conditions it is impossible that an object’s retinal size stays constant across depth planes. According to the principles of size constancy scaling, increasing the offset between near and far target location is expected to increases the amount of conflicting information. This in turn may have prevented the effect of different depth positions to arise in this experimental condition. Seemingly, there is also a non-significant trend in the data indicating a switch of RT related to both retinal size conditions across experiments. Stimuli in *retinal size constant* condition elicited faster RT in Experiment 1, while in Experiment 2 faster RT were observed in the *retinal size variable* condition. This effect may also be related to the fact that conflicting information is more pronounced in Experiment 2 in the *retinal size constant* condition. However, this trend again is difficult to assess as it is derived from different participant groups. Finally, it has to be noted that in the *retinal size variable* condition the physical size differences between near and far target become more pronounced if offset is increased. Even though it is unlikely that the reported effect solely originates from these differences in physical size, a partial contribution cannot completely be ruled out.

### Target Type Modulation

The influence of familiar objects on visual processing under reduced viewing conditions has been extensively discussed in past (e.g., Schiffman, 1967; Gogel and Da Silva, 1987; Predebon, 1994). There is agreement that familiar objects operate as determinant for perceived size and distance. In line with that, Sperandio et al. (2009) reported that the inverse relationship between RT and perceived size was restricted to familiar object stimuli. According to the authors presenting familiar objects can lead to a more efficient operating of size constancy mechanisms. Thus, this prediction was also tested in the present study using two different target types (i.e., plain sphere vs. soccer ball). The results from both experiments did not suggest a strong relationship between RT and familiar objects. However, in Experiment 2 there was a significant RF effect observable, indicating less intense responses associated with ball targets (and there was also an analog non-significant trend in RT data pointing toward slower responses elicited by ball targets). Since this effect is only evident in Experiment 2 it is unlikely that it originates from differences in stimulus intensity. Assuming that familiar objects (such as the ball target used in the present experiments) require more elaborated size constancy scaling a larger offset between two target sites should add additional demands with respect to these scaling processes. As described above the offset between near and far target locations was larger in Experiment 2 which in turn resulted in stronger deviations of size between both target positions. Yet, there is no marked difference between ball and sphere target with respect to the modulation of their perceived depth, as would be predicted from the findings of Sperandio et al. (2009). Even though the impact of target type was increased in Experiment 2 no such interactive influence of the ball target in this stereoscopic experimental setting was observed. However, in contrast to the experiments by Sperandio and colleagues target type was varied within rather than between experimental blocks in the present experiments. Given that both targets were presented as 3D objects which only differed in terms of texture the spatial representation of the ball could have been easily transferred to the sphere and thus its impact was potentially diminished.

## Conclusion

Taken together the present study provides strong support for the idea that within virtual 3D environments there is a body-centered spatial (search) gradient (Finlayson and Grove, 2015; Plewan and Rinkenauer, 2016). Irrespective of perceived size modulation participants reacted faster and more forcefully toward objects presented closer to the observer. Previous research revealed that perceived object size constitutes a major determinant of RT (Sperandio et al., 2009, 2010; Plewan et al., 2012; Savazzi et al., 2012). In a recent review, it has been outlined that depth perception within virtual 3D environments can substantially differ from perception in the real world (Renner et al., 2013). This can account for the stable differences between studies modulating perceived size in virtual 3D space and those using conventional stimulus material. Furthermore, the present results elucidate that even subtle differences in experimental designs can lead to different inferences on 3D processing. Finally, on a theoretical level, the results suggest that (at least under these viewing conditions) objects perceived as closer most likely are associated with a higher behavioral urgency and thus elicit faster and more forceful responses.

## Ethics Statement

This study was carried out in accordance with the recommendations of the Ethics Committee of the Leibniz Research Centre for Working Environments and Human Factors with written informed consent from all subjects. All subjects gave written informed consent in accordance with the Declaration of Helsinki. The protocol was approved by the Ethics Committee of the Leibniz Research Centre for Working Environments and Human Factors.

## Author Contributions

TP was responsible for study design, data acquisition, analysis, interpretation, and wrote the paper. GR was responsible for study design, data interpretation, and wrote the paper. TP and GR approved the final version of the manuscript.

## Conflict of Interest Statement

The authors declare that the research was conducted in the absence of any commercial or financial relationships that could be construed as a potential conflict of interest.
